# Case report: Single-cell mapping of peripheral blood mononuclear cells from a patient with both Crohn’s disease and isolated congenital asplenia

**DOI:** 10.3389/fimmu.2022.959281

**Published:** 2022-08-26

**Authors:** Dan Pu, Lu Liu, Na Wang, Dandan Wang, Zhe Zhang, Baisui Feng

**Affiliations:** ^1^ Department of Gastroenterology, The Second Affiliated Hospital of Zhengzhou University, Zhengzhou, China; ^2^ Department of Microbiology and Immunology, School of Basic Medical Sciences, Zhengzhou University, Zhengzhou, China

**Keywords:** CyTOF, Crohn’s disease, isolated congenital asplenia, PBMC, spleen

## Abstract

Crohn’s disease (CD), as one of the principal form of inflammatory bowel disease (IBD), is characterized by the chronic and recurring inflammatory conditions in the intestine resulting from the over-activation of intestinal immunity. Hyposplenism is strongly associated with CD, while the effect of human spleen on the differentiation and development of immune cell subsets in CD patients remains unclear. Isolated congenital asplenia (ICA) is an extremely rare condition characterized by the absence of a spleen at birth without any other developmental defects. Here, we describe the first case of a patient with both ICA and CD, and follow the progression of CD from remission to active stage. Using cytometry by time of flight (CyTOF) analysis, we draw the first single-cell mapping of peripheral blood mononuclear cells (PBMC) from this unique patient, tracing back to the innate or adaptive immune cell subsets and cell surface markers affected by the spleen. Based on our analysis, it is speculated that the spleen contributes to maintaining immune homeostasis, alleviating intestinal inflammation and improving prognosis by influencing the differentiation and development of peripheral immune cell subsets and the expression of cell surface markers in patients with CD.

## Introduction

Hyposplenism is related to the development of various diseases (1–3). Inflammatory bowel disease (IBD), a group of chronic immune-mediated intestinal inflammation diseases that includes Crohn’s disease (CD), has been found to be complicated by hyposplenism for more than 30 years (4, 5). But to date, the impact of spleen on the alteration of peripheral immune components in CD patients remains unclear. Many animal studies have demonstrated the role of mouse spleen on the development of immune cell subsets (6, 7), but due to various ethical issues, studies on the human spleen are fraught with challenges. Here, we report the first case of a patient with both CD and isolated congenital asplenia (ICA), and focus on alterations in circulating immune cell subsets and cell surface markers, with the aim of investigating the effect of human spleen on the differentiation and development of peripheral immune cells, exploring its potential impact on immune homeostasis in CD, and providing evidences for future studies on the role of human spleen in peripheral immunity of CD.

## Case presentation

**Figure 1:**
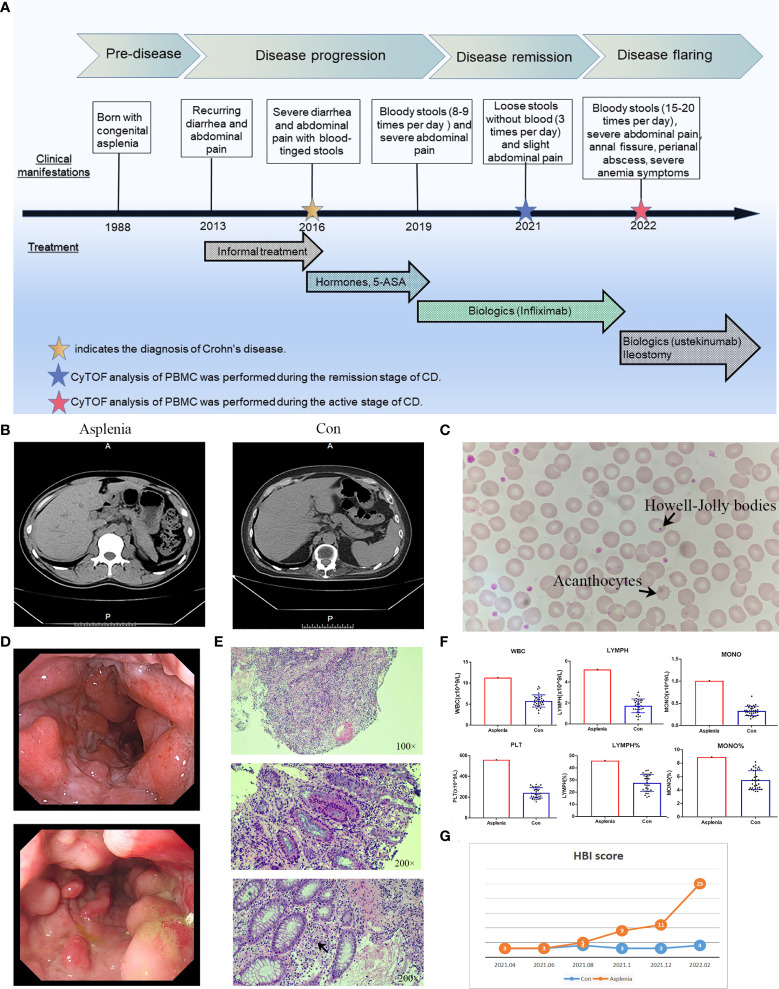
Clinical characteristics in the patient with both ICA and CD. **(A)** Timeline of the clinical events and treatment strategies. **(B)** Abdominal CT images of the congenitally asplenic patient (left) and the control (right). **(C)** Morphological examination of peripheral blood smear of the congenitally asplenic patient showed the acanthocytes and Howell-Jolly bodies within red blood cells (arrows). **(D)** Colonoscopy demonstrating multiple longitudinal deep ulcers with mucosal oedema and cobblestone appearance in the hepatic flexure (top panel) and the sigmoid colon (bottom panel) of the asplenic CD patient. **(E)** Hematoxylin-eosin staining of the colonoscopic biopsy tissue from the asplenic CD patient showing the infiltration of lymphocytes, plasma cells and neutrophils in the lamina propria of the mucosa, discontinuous severe crypt distortion (top panel) as well as the ulcer (middle panel) and crypt abscess (bottom panel, arrow) formation. **(F)** The histograms demonstrating the elevated WBC (white blood cell count), PLT (platelet count), LYMPH (absolute number of lymphocytes), LYMPH% (percentage of lymphocytes), MONO (absolute number of monocytes) and MONO% (percentage of monocytes) of the asplenic CD patient in remission compared to remission controls (n=32) with normal spleens randomly selected from our center’s CD cohort. **(G)** Line graph showing the difference in disease progression between the asplenic patient and the control after the first CyTOF analysis (HBI score used to evaluate disease activity of CD).

The patient, a 33-year-old man, has been suffering from CD for 9 years (**Figure 1A**). Born with the absence of spleen, he was diagnosed with non-familial type isolated congenital asplenia (ICA) after other developmental defects and family history were excluded (**Figure 1B**) (8). On the peripheral blood smear of this patient, acanthocytes and Howell-Jolly bodies within red blood cells were detected, suggesting the defect of spleen phagocytic function (**Figure 1C**). In consequence, he was susceptible to various infections, such as respiratory and digestive infections, especially in his early years. In 2013, recurrent abdominal pain and diarrhea developed, which was not taken seriously at first and no medication was administered. As his clinical manifestations gradually worsened, blood-tinged stools appeared in 2016. After colonoscopy and pathology tests (**Figures 1D, E**), he was definitively diagnosed with Crohn’s disease and accepted the treatment with prednisone, thalidomide and mesalazine. However, the symptoms were not alleviated but aggravated in 2019. Infliximab, a monoclonal antibody binding to tumor necrosis factor-alpha (TNF-alpha), was then administered at the dose of 6 mg/kg, and his symptoms subsequently improved. Within the first 1.5 years of infliximab therapy, the patient showed a good response with significant relief from recurrent bloody mucopurulent stools and abdominal pain.

During the remission stage of CD, when the Harvey Bradshaw Index (HBI) score was 3, the noticeable increase in the absolute numbers of peripheral blood leucocytes, monocytes, lymphocytes and platelets were observed in this asplenic patient compared to other CD patients in remission with normal spleens, as were the percentages of monocytes and lymphocytes (**Figure 1F**). Therefore, it was of great interest which immune cell subsets or surface markers were altered in the PBMC from this asplenic patient and the potential impact of these changes on peripheral immunity on CD. To facilitate comparison, another CD patient with a normal spleen who had the same sex (male), disease phenotype (Montreal classification, A2L3B1) and medication history (prednisone, thalidomide, mesalazine and infliximab), as well as very similar age (34-year-old), medical history (9 years of CD) and clinical manifestations, was selected as the optimal control for the more in-depth study, namely cytometry by time of flight (CyTOF) (**Figure 1B**). Unlike Fluorescence Activated Cell Sorting (FACS) commonly employed in laboratories, CyTOF uses transition element isotopes instead of fluorophores as markers for antibodies and analyses these isotopes simultaneously by a time-of-flight mass spectrometer, enabling high-dimensional single-cell analysis without compensating for spectral overlap (9). Subsequently, CyTOF analysis was performed on the peripheral blood mononuclear cells (PBMC) from these two patients. (**Supplementary Material Data Sheet 1**; **Supplementary Figure 1**)

After the CyTOF analysis, the prognosis of these two CD patients was followed up. It was found in amazement that the disease severity of this asplenic patient altered significantly during the following 1 year compared with the control (**Figure 1G**). In October 2021, a perianal abscess developed and the frequency of defecation increased gradually. In December 2021, mucopurulent stools and abdominal pain recurred. By February 2022, ustekinumab was administrated due to the secondary loss of response to infliximab. Unfortunately, ustekinumab failed to show the expected efficacy. Meanwhile, multiple longitudinal ulcerations with pus and blood in the the patient’s colon was detected by colonoscopy, as well as the combination of significant anal fissures and perianal abscesses. Therefore, the patient received ileostomy. The timeline of this case is showed in **Figure 1A**.

**Figure 2:**
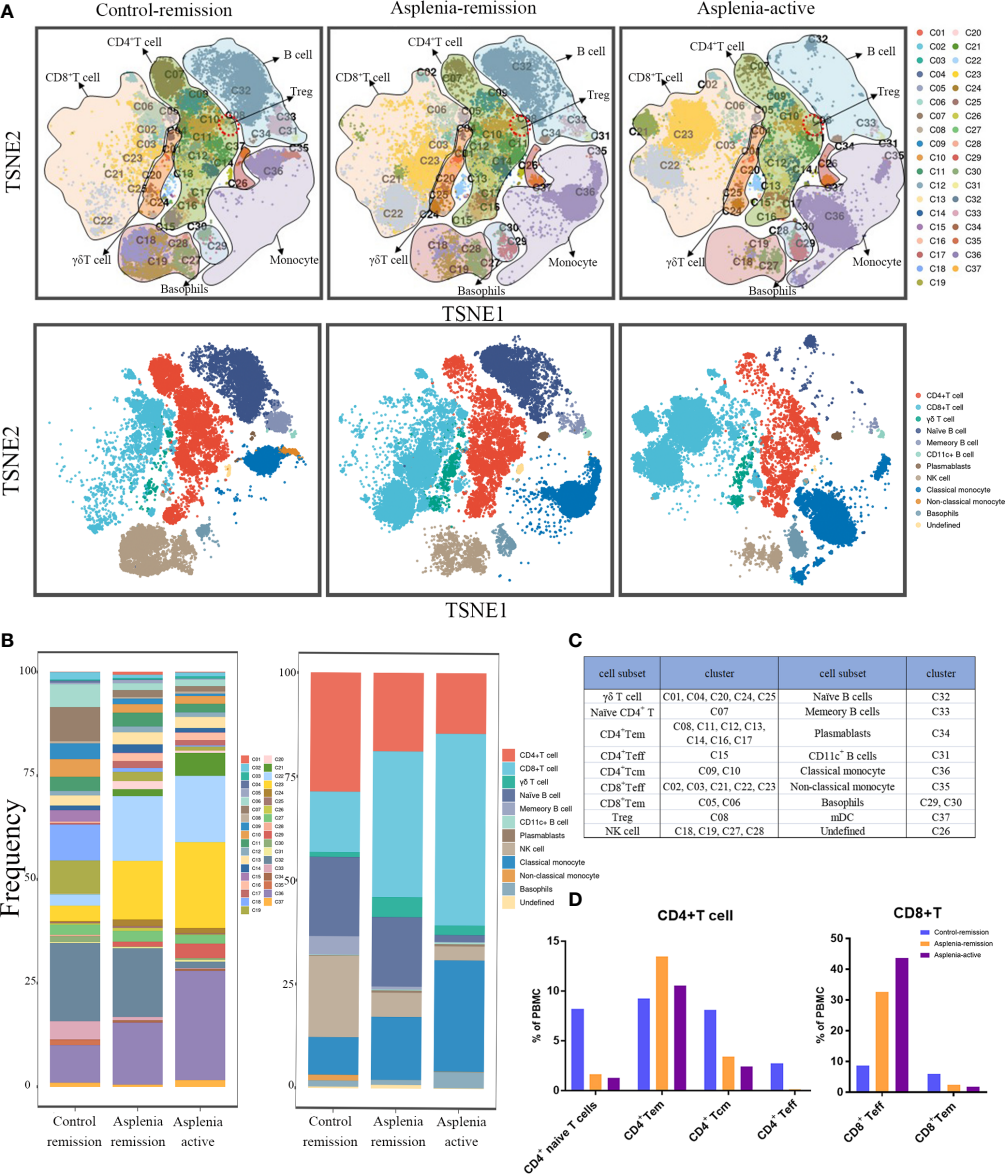
Single-cell mapping of peripheral blood mononuclear cells showed the significant differences of immune cell subsets among the control patient in remission and the asplenic patient in remission or active stage of CD. **(A)** Mass cytometric analysis and mapping of PBMC from the control patient in remission (left), the asplenic patient in remission (medial) and the asplenic patient in active stage of CD (right) using Cytometry by Time Of Flight (CyTOF) mass spectroscopy, t-distributed stochastic neighbor embedding (t-SNE) and clustering algorithms. (Upper panel: mapping of PBMC into 37 clusters based on differences in cell surface marker expression; lower panel: depicting differences in the distribution of cell subsets in PBMC based on 37 clusters.) **(B)** Bar graphs showing the difference in frequency of each cluster (left panel) or cell subset (right panel) across different samples. **(C)** Annotation of the immune cell subsets based on different clusters. **(D)** Differences in the frequency of each subset of CD4^+^ T cells and CD8^+^ T cells among the different samples. (Tem, effector memory T cells; Tcm, central memory T cells; Teff, effector T cells).

**Figure 3:**
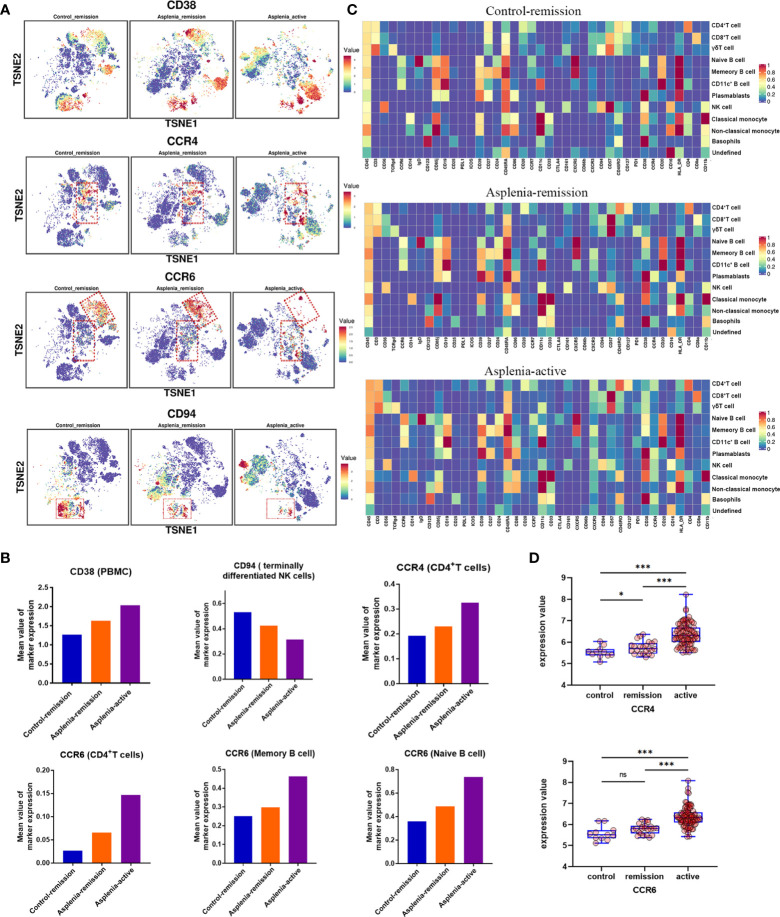
High-dimensional analysis revealing the differences in the expression of specific surface markers on PBMC samples from the control patient and congenitally asplenic patient with remission CD, as well as the congenitally asplenic patient in the active stage of CD. **(A)** The t-distributed stochastic neighbor embedding plots colored by expression of CD38, CCR4, CCR6 and CD94 of each group. **(B)** The histograms displaying differences in the expression of surface markers (CD38, CCR4, CCR6 and CD94) across patient samples. **(C)** Heatmap representing the marker expression profiles of the 12 cell populations in PBMC. The heatmap colors represent the average expression of a given marker for each cell population. **(D)** Boxplot showing the difference in the expression of CCR4 (top) and CCR6 (bottom) in colon tissues from healthy controls, remission IBD and active IBD patients, with differences assessed by the GEO2R bioinformatics tool. (Raw data from GEO database, GSE59071; ns, not significant; *P<0.05; ***P<0.001).

During his active stage of CD, CyTOF was utilized again to characterize alterations in circulating immune cells. The PhenoGraph clustering algorithm was then applied to all samples from the control and asplenic patient in remission, as well as the asplenic patient in active stage for comparative analysis (**Figures 2**, **3**).

In terms of innate immunity, a significant decrease of non-classical monocytes (cluster 35) was observed in the asplenic patient in remission compared with the control, while classical monocytes (cluster 36) showed a relative elevation, accompanied by an upregulation of CD38. These alterations were more pronounced during the active stage of CD (**Figures 2A, B**; **Figures 3A, B**). The frequency of natural killer (NK) cells, especially mature types (cluster 18,19, 28), reduced considerably in the asplenic patient in remission, and were further minimized during the active stage. Meanwhile, compared with the control, down-regulated expression of the inhibitory receptor CD94 in clusters 18 and 19, which represent the terminally differentiated CD57^+^ NK cells, was noticeable in the asplenic patient during disease remission, which became more obvious as the disease progressed (**Figures 3A, C**). Interestingly, while the frequency of NK cells decreased, the expression of CD38 was significantly upregulated both in the active and remission stage of the asplenic patient.

As for the adaptive immune system, in the T lymphocyte population, elevated CD8^+^ T cells, predominantly CD8^+^ effector T cells (cluster 21, 22, 23), were identified in the asplenic patient in remission compared with the control, and reached its highest enrichment in the active period (**Figures 2A, B, D**). In contrast, the frequency of CD4^+^ T cells, including naïve T cells (cluster 07), effector T cells (Teff, cluster 15) and central memory T cells (Tcm, cluster 09,10), was reduced in the asplenic patient in remission. And as the disease progressed, the frequency of circulating CD4^+^ T cells, including effector memory T cells (Tem), decreased distinctly during the active phase of CD. Therefore, the CD4^+^/CD8^+^ T-cell ratio was remarkably lower than normal (range: 1.5 to 2.5) in the asplenic patient compared with the control in remission (10). Meanwhile, the imbalance in T-cell homeostasis became more severe during the active CD (**Figures 2A, B**). More interestingly, CD4^+^ T cells from the asplenic patient showed higher expression of CCR4 and CCR6, and their expression levels increased progressively with disease severity, which was not observed on CD8^+^ T cells (**Figures 3A–C**). In addition, the frequency of regulatory T cells (Treg, cluster 08) did not show significant differences in all samples.

Among B lymphocytes, although the frequency of naïve B cells (cluster 32) was mildly reduced in the asplenic patient in remission, the expression level of CCR6 was higher on these cells than the control (**Figures 3A, B**). Furthermore, compared with the remission phase, the asplenic patient with active CD showed a dramatic reduction in circulating naïve B cells accompanied by the highest expression of CCR6. Similarly, memory B cells (cluster 33) reduced in the asplenic patient, but the expression of CCR6 was upregulated, which was more pronounced in the active stage (**Figure 3B**). Meanwhile, plasmablasts (cluster 34) also showed a tendency to be elevated in the asplenic patient (**Figures 2A, B**).

**Figure 4:**
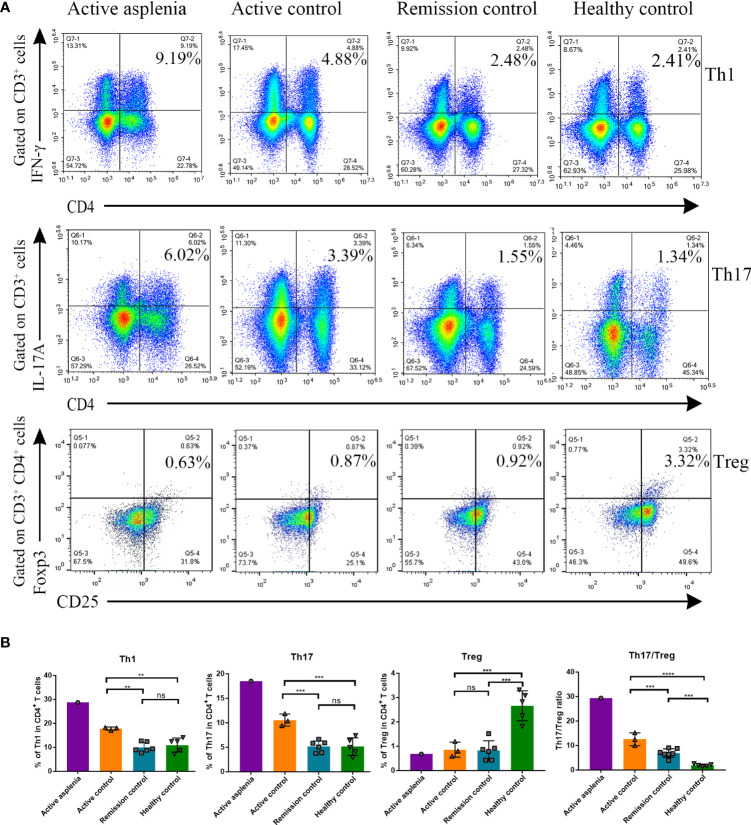
Flow cytometry analysis demonstrating the differences in proportions of Th1, Th17 and Treg cells from PBMC and the Th17/Treg ratio among healthy controls, remission controls, active controls and the congenitally asplenic patient with active CD. **(A)** Representative FACS plots showing the percentage of CD3^+^CD4^+^IFN-γ^+^ (Th1) cells (top panel: gated on CD3^+^ cells), CD3^+^CD4^+^IL-17A^+^ (Th17) cells (middle panel: gated on CD3^+^ cells) and CD3^+^CD4^+^CD25^+^Foxp3^+^ (Treg) cells (bottom panel: gated on CD3^+^CD4^+^ cells) of each group [asplenia with active CD (n=1), controls with active CD (n=3), controls with remission CD (n=6), healthy controls (n=5)]. **(B)** The histograms showing the difference in the percentage of Th1, Th17 and Treg cells in CD4^+^ T cell, as well as the Th17/Treg ratio. Data were analyzed by One-way ANOVA, and all values are reported as means and SD. ns, not significant; **P < 0.01; ***P < 0.001; ****P < 0.0001.

Along with CyTOF analysis, we also performed multicolor flow cytometry for Th1, Th17 and Treg cells from the asplenic patient during his active stage, considering the essential role of these cells in the pathogenesis of CD. Patients with an active or remitting CD and healthy individuals were selected as controls (**Figure 4**; **Supplementary Figure 2**). The analysis demonstrated that the percentage of Th1 and Th17 cells and the Th17/Treg ratio increased gradually with disease progression in CD patients, but this was not observed in Treg cells, consistent with the result of CyTOF analysis in the asplenic patient. Notably, despite the similar disease activity scores, Th1 and Th17 cells were significantly enriched in PBMC from the asplenic patient compared to other active CD patients, accompanied by a dramatic increase in the ratio of Th17/Treg cells (**Figures 4A, B**).

## Diagnostic assessment

The two patients were diagnosed and treated according to the British Society of Gastroenterology consensus guidelines (11). The diagnosis of Crohn’s disease was confirmed by the patient’s medical history, clinical symptoms, ileoscopy with biospy histology, CT enterography and laboratory tests. The Harvey Bradshaw Index (HBI) is employed to assess the activity of the disease (12). PBMC samples in remission stage of CD were collected from the asplenic patient and the control both with an HBI score of 3. During ileostomy treatment (HBI score of 25), the asplenic patient provided the active PBMC sample.

## Discussion

CD, characterized by an over-activation of intestinal immunity, often accompanied by impaired splenic function (13). Although the relationship between CD and hyposplenism has not been well characterized, there is no doubt that the spleen, as the largest peripheral immune organ in the body, plays an essential role in shaping the peripheral immune components, which may further alters the immune environment of the intestine. In our case, using CyTOF, we draw the first single-cell landscape of PBMC from the congenitally asplenic patient during both remission and active stages of CD, and characterize the immune cell subsets and functional cell surface markers of PBMC affected by human spleen, further exploring potential targets for the treatment of CD.

It has been well-accepted that peripheral blood monocytes are divided into three different subsets: classical, intermediate, and nonclassical monocytes (14). Classical monocytes, which secrete various pro-inflammatory cytokines when activated, can also develop into non-classical monocytes to perform protective functions (15, 16). Non-classical monocytes, the progenitors of intestinal wound healing macrophages, mediate the mucosal healing after homing to the gut *via* α4β7 integrin (17). Research on the transformation of classical to non-classical monocytes has been a hot topic in recent years, but most studies are currently conducted *in vitro* or in animal experiments. Given the marked decrease of non-classical monocytes and increase of classical monocytes in PBMC of the asplenic patient, it seems that human spleen contributes to the differentiation of classical monocytes to non-classical types, further mediating the healing of intestinal mucosa, as confirmed in mice (6).

Natural killer (NK) cells play an essential role in the regulation of innate and adaptive immunity, and its dysregulation is associated with the pathogenesis of CD (18, 19). In contrast to tissue-resident molecular signatures of immature NK cells (CD56^bright^CD16^-^), mature NK cells mediate peripheral immune surveillance against pathogen invasion (20). In PBMC of the asplenic patient with perianal abscesses, the frequency of mature NK cells was reduced significantly, further confirming the preventive role of mature NK cells in secondary infections associated with CD. Moreover, in the remission stage, mature NK cells from the asplenic patient are markedly reduced compared with the control, suggesting that the human spleen is another important site for the maturation of NK cells in addition to the bone marrow, which is consistent with the results of animal and *in vitro* experiments (7, 21). Consequently, it is hypothesized that the spleen contributes to improve immune surveillance by promoting the maturation of NK cells, thereby reducing the risk of secondary infection and improving the prognosis of CD.

In addition, the activity of NK cells, which is defined by the integration of signals from inhibitory and activating receptors, correlates with the pathogenesis of CD (22). The over-activated circulating mature NK cells produce large amounts of pro-inflammatory cytokines, such as IFN-γ, TNF and IL-17A, increasing their cytotoxicity and promoting the activation and polarization of other immune cells (18, 23). Interestingly, as an important inhibitory receptor for NK cells, CD94 provides educational signal to downregulate the production of pro-inflammatory cytokines and eliminate activated NK cells to prevent excessive inflammation (24). Researchers have demonstrated that reduced CD94 expression on NK cell is strongly associated with persistent inflammation and poor prognosis in other chronic inflammatory diseases (25, 26). Based on our results, the expression of CD94 on mature NK cells from the asplenic patient was significantly reduced. Therefore, it is conceivable that human spleen not only promotes the maturation of NK cells to perform immunosurveillance functions, but also suppresses NK cell-mediated inflammatory cascade response by regulating CD94 expression, which contributes to the maintenance of immune homeostasis in CD patients.

In adaptive immunity, imbalance of the CD4^+^/CD8^+^ T-cell ratio, a marker of immune function, has been observed in a variety of immune-mediated inflammatory diseases (10, 27). In this study, the CD4^+^/CD8^+^ T-cell ratio in the PBMC of this asplenic patient gradually decreased as the disease worsened, highlighting the association between the CD4^+^/CD8^+^ T-cell ratio of PBMC and disease severity. Interestingly, during the remission stage of CD, despite sharing the same disease activity score, the CD4^+^/CD8^+^ T-cells ratio of the asplenic patient decreased obviously compared with the control, suggesting that the asplenic patient in remission experienced more severe immune disorder and a worse prognostic risk, which was also confirmed by the subsequent disease progression of this patient.

To explore the potential mechanisms of the spleen affecting the CD4^+^/CD8^+^ T-cell ratio in CD, we focused on the differential expression of functional cell surface markers on T lymphocytes. Consistent with the results of a recent high-dimensional analysis (28), the upregulation of CCR4 and CCR6 expression in circulating T lymphocytes from the asplenic patient was observed. And our study further revealed that CD4^+^ T cells from the asplenic patient expressed higher CCR4 and CCR6 than CD8^+^ T cells, especially in the active stage of CD. It is well known that during intestinal inflammation, epithelial cells and immune cells in the intestine secrete different chemokines (e.g. CCL20 and CCL17) and recruit immune cells from peripheral blood to participate in the adaptive immune response in the gut through receptor-ligand interaction (29, 30). The migration of circulating immune cells to the inflamed gut mediated by the chemokine axis CCL20-CCR6 has been revealed in many previous studies of CD (31, 32). Moreover, recent animal studies have also highlighted the key role of the chemokine axis CCL17-CCR4 in the pathogenesis of colitis (33, 34). In addition, using database analysis (**Figure 3D**), we demonstrated that the expression of CCR6 and CCR4 in the intestinal mucosa was strongly correlated with the severity of IBD, as confirmed in the Genome-wide Association Studies (GWAS) (35). Consequently, we propose that the enhanced migration of CD4^+^ T cells with high expression of CCR6 and CCR4 to the inflamed intestinal mucosa results in the unbalanced CD4^+^/CD8^+^T-cell ratio. This is consistent with the latest findings, which highlight that it is CD4^+^ T cells but not CD8^+^ T cells that are markedly increased in the intestinal mucosa of active CD (36). Considering the elevated expression of CCR6 and CCR4 on the surface of CD4^+^ T cells and the reduced CD4^+^/CD8^+^T-cell ratio in the asplenic patient compared with the control in remission, it is hypothesized that the spleen may regulate the migration of CD4^+^ T cells to the intestine by modulating the expression of CCR6 and CCR4, thereby improving the prognosis of CD.

Among CD4^+^ T lymphocytes, we focused on several key subpopulations, Th1, Th17, and Treg cells, which were proven to be strongly associated with the pathogenesis of CD. As is showed in **Figure 4**, Th1 and Th17 cells, which correlate with disease severity, were visibly enriched in the PBMC of the asplenic patient compared with other patients with active CD. Moreover, although no difference was found in the percentage of Treg cells among the CD groups, the ratio between Th17/Treg cells was significantly higher in the asplenic patient than others. Given that the regulation of Th17/Treg cell balance is a potential new strategy of CD treatment (37), it is conjectured that human spleen may reduce intestinal inflammation and improve the prognosis by regulating the Th17/Treg ratio, as well as percentages of Th1 and Th17 cells.

Despite the predominance of research on T lymphocytes in the pathogenesis and treatment of CD, emerging evidence suggests that the role of B cells cannot be overlooked as well. Early studies have demonstrated that the decrease in memory B cells and increase in plasmablasts from PBMC of CD patients are negatively associated with disease remission (38). This corresponded to the alterations in memory B cells and plasmablasts observed in the asplenic patient in remission. In addition, recent studies have revealed that memory B cells and naïve B cells are abundantly enriched in the gut-associated lymphatic tissue (GALT) and intestinal mucosa of patients with CD (39, 40). Therefore, we speculate that high expression of CCR6 on both naive B cells and memory B cells in the asplenic patient leads to increased migration of these cell subsets to the intestinal mucosa, whereas the spleen can downregulate this process by reducing the expression of CCR6, thereby alleviating intestinal inflammation and improving prognosis.

Along with the regulation of chemokines, the expression of CD38 on PBMC was also regulated by the spleen. CD38, a molecule widely expressed on immune cells, is frequently used as a hallmark of cell activation (41). Due to its ability to regulate multiple components of the inflammatory process, such as cell migration, activation, antigen presentation and cytokine release, CD38 is thought to be closely associated with chronic inflammation and autoimmunity (42). Expression of CD38 on intestinal inflammatory cells and its involvement in the NAD metabolic pathway have been proved to promote colitis, whereas reduced expression levels and activity of CD38 correlates with recovery from intestinal inflammation (43, 44). According to our findings, the expression of CD38 on PBMC from the asplenic patient was higher in the active stage of CD than in remission, highlighting the correlation between the CD38 and gut inflammation. Furthermore, given the upregulation of CD38 on PBMC from the asplenic patient compared with the control in remission, it is hypothesized that the spleen may antagonize intestinal inflammation by regulating the expression of CD38 on peripheral immune cells.

## Conclusion

In summary, we report the first case of a patient with both Crohn’s disease and isolated congenital asplenia, and draw the first landscape of peripheral blood mononuclear cells from this unique patient during the remission and active stages of CD, which provide valuable information for studying the role of spleen on the differentiation and development of circulating immune cells in CD. We propose that human spleen contributes to maintaining immune homeostasis, alleviating intestinal inflammation and improving prognosis by regulating the differentiation and proportion of specific immune cell subsets, as well as the expression of certain functional cell surface markers such as CCR6 and CCR4, all of which may be the potential therapeutic targets for CD. Furthermore, targeting the function of human spleen will open a new paradigm in immunomodulatory therapy, which may replace the traditional concept of CD treatment based on immunosuppression. The cytometry technique of CyTOF used in this case provided detailed subsets of PBMC from the special patient based on cell markers, which is very useful to study the effect of spleen on different immune cell subsets at single-cell resolution. Although the sample size was insufficient due to the extreme rarity of this group of patients, based on the present results, it is of great value to focus on the function of the human spleen in the research and treatment of CD. Future studies will incorporate animal experiments to delve into mechanistic issues.

## Patient perspective

The patient is currently recovering from the ileostomy. Considering the complexity of the disease caused by congenital asplenia, he will stay in close contact with us to respond to the various potential risks of infection and blood clots in a timely manner.

## Data availability statement

The raw data supporting the conclusions of this article will be made available by the corresponding authors, without undue reservation.

## Ethics statement

The studies involving human participants were reviewed and approved by ethics committee of the Second Affiliated Hospital of Zhengzhou University. The patients/participants provided their written informed consent to participate in this study. Written informed consent was obtained from the individual(s) for the publication of any potentially identifiable images or data included in this article.

## Author contributions

ZZ and BF designed the study, provided supervision. DP performed experiments, analyzed data, and wrote the manuscript. DP, LL, and DW participated in the clinical care and management of patients. BF, NW, and DW provided funding, conceptual advice, discussed results, and critically revised the manuscript. All authors contributed to the article and approved the submitted version.

## Funding

The work was supported by the Medical Science and Technology Breakthrough Projects of Henan Province, China (No.LHGJ20190327), the Specialized Project for Frontier Cross Research of Zhengzhou University (No. XKZDQY201906) and the Key R&D and Promotion Projects of Henan Province, China (Technology Research; No. 212102310803).

## Acknowledgments

We thank the patient and her families for providing permission to share the medical information. We thank the physicians for offering professional consultations. We are grateful to other members of the Inflammatory Bowel Disease Study Group for providing materials for this study.

## Conflict of interest

The authors declare that the research was conducted in the absence of any commercial or financial relationships that could be construed as a potential conflict of interest.

## Publisher’s note

All claims expressed in this article are solely those of the authors and do not necessarily represent those of their affiliated organizations, or those of the publisher, the editors and the reviewers. Any product that may be evaluated in this article, or claim that may be made by its manufacturer, is not guaranteed or endorsed by the publisher.

## Supplementary material

Supplementary Figure 1 Gating strategy for mass cytometry by time of flight (CyTOF).

Supplementary Figure 2 Gating strategy for multicolor flow cytometry.
