# Supplementary figures and images for: Case report: Single-cell mapping of peripheral blood mononuclear cells from a patient with both Crohn’s disease and isolated congenital asplenia
Source: Front Immunol. 2022 Aug 26;13:959281. doi: 10.3389/fimmu.2022.959281 (PMC9459022; doi:10.3389/fimmu.2022.959281)

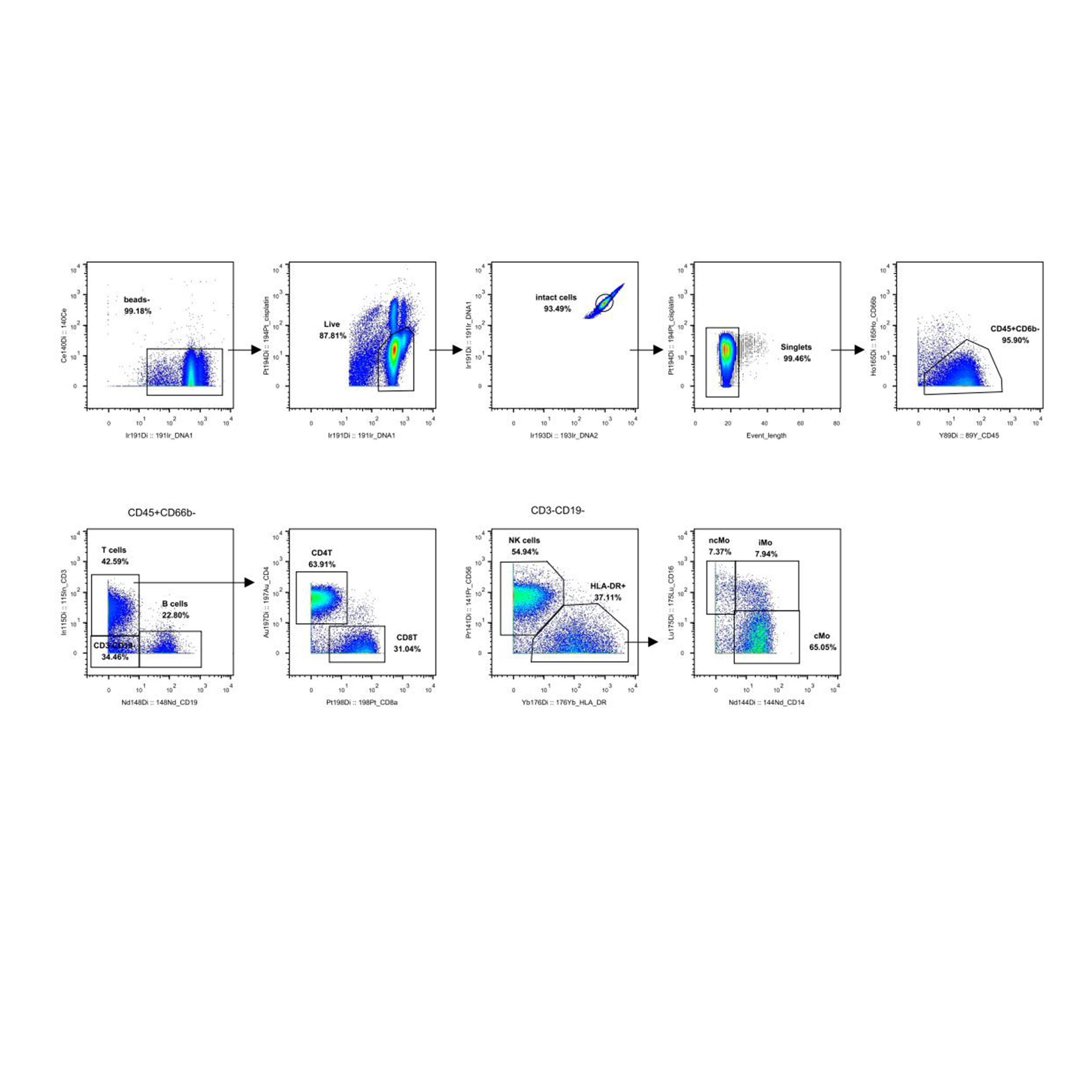

Supplement: Supplementary Figure 1 — Gating strategy for mass cytometry by time of flight (CyTOF). [file Image_1.tif]

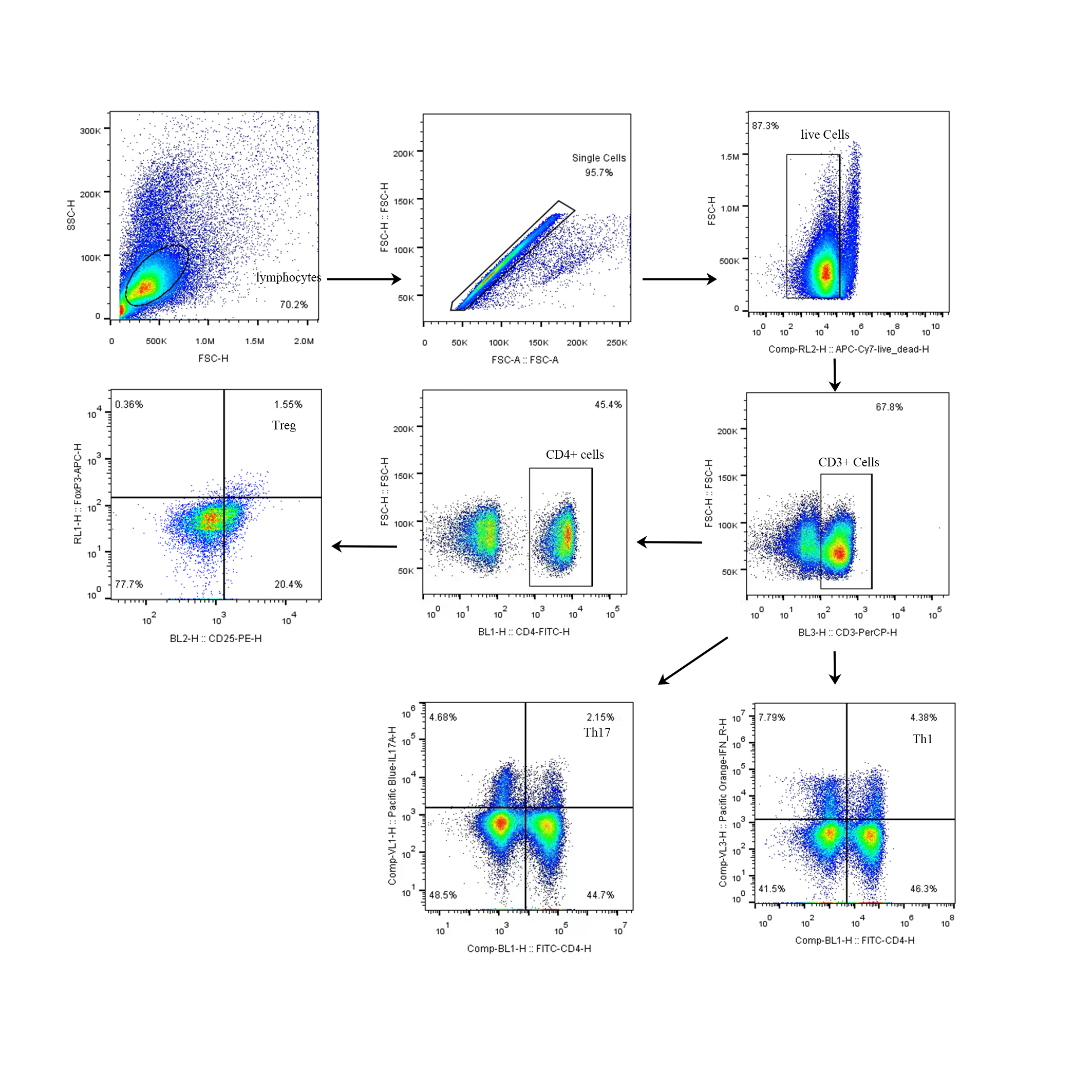

Supplement: Supplementary Figure 2 — Gating strategy for multicolor flow cytometry. [file Image_2.tif]
