# Supplementary material for: Case report: Single-cell mapping of peripheral blood mononuclear cells from a patient with both Crohn’s disease and isolated congenital asplenia
Source: Front Immunol. 2022 Aug 26;13:959281. doi: 10.3389/fimmu.2022.959281 (PMC9459022; doi:10.3389/fimmu.2022.959281)
Supplement: Supplementary file 3 [file DataSheet_1.docx]

Supplementary Material

# 1 Supplementary Table 1

# Representative markers for each cluster of immune cell subsets from peripheral blood

| **Cluster ID** | **Cell subset** | **Marker expression of each cluster** |
| --- | --- | --- |
| C01 | γδT cells | CD3^+^CD4^-^CD8a^+^CD27^+^CD57^+^PD1^+^HLA-DR^mid^ |
| C02 | CD8^+^ effector T cells | CD3^+^CD8a^+^CD45RA^+^CCR7^-^CD27^+^CD57^-^ |
| C03 | CD8^+^ effector T cells | CD3^+^CD8a^+^CD45RA^+^CCR7^-^ CD27^-^CD57^-^ |
| C04 | γδT cells | CD3^+^CD4^-^CD8a^-^CD94^+^CD57^-^ |
| C05 | CD8^+^ effector memory T cells | CD3^+^CD8a^+^CD45RA^-^CCR7^-^CD27^+^CD161^+^ |
| C06 | CD8^+^ effector memory T cells | CD3^+^CD8a^+^CD45RA^-^CCR7^-^CD27^+^CD57^-^ |
| C07 | CD4^+^ naive T cells | CD3^+^CD4^+^CD45RA^+^CCR7^+^CD27^+^ |
| C08 | CD4^+^ effector memory T cells | CD3^+^CD4^+^CD45RA^-^CCR7^-^CD25^+^CD127^-^ICOS^mid^CD39^+^CD27^+^CCR4^+^HLA-DR^mid^ |
| C09 | CD4^+^ central memory T cells | CD3^+^CD4^+^CD45RA^-^CCR7^+^CXCR5^+^CCR4^-^ |
| C10 | CD4^+^ central memory T cells | CD3^+^CD4^+^CD45RA^-^CCR7^+^CXCR5^-^CCR4^+^CD27^+^ |
| C11 | CD4^+^ effector memory T cells | CD3^+^CD4^+^CD45RA^-^CCR7^-^CXCR3^+^CCR4^-^ |
| C12 | CD4^+^ effector memory T cells | CD3^+^CD4^+^CD45RA^-^CCR7^-^CCR4^+^CD27^-^CD28^+^ |
| C13 | CD4^+^ effector memory T cells | CD3^+^CD4^+^CD45RA^-^CCR7^-^CD28^+^CD57^-^ |
| C14 | CD4^+^ effector memory T cells | CD3^+^CD4^+^CD45RA^-^CCR7^-^CD28^+^CD161^+^ |
| C15 | CD4^+^ effector T cells | CD3^+^CD4^+^CD45RA^+^CCR7^-^CD56^+^CD57^+^ |
| C16 | CD4^+^ effector memory T cells | CD3^+^CD4^+^CD45RA^-^CCR7^-^CD56^-^CD57^+^ |
| C17 | CD4^+^ effector memory T cells | CD3^+^CD4^+^CD45RA^-^CCR7^-^CD28^+^CD57^+^ |
| C18 | NK cells | CD56^+^CD16^+^CD11b^+^CD11c^-^CD57^+^CD38^+^ |
| C19 | NK cells | CD56^+^CD16^+^CD11b^+^CD11c^+^CD57^+^CD38^+^ |
| C20 | γδT cells | CD3^+^CD4^-^CD8a^+^CD27^+^CD57^-^PD1^+^HLA-DR^mid^ |
| C21 | CD8^+^ effector T cells | CD3^+^CD8a^+^CD45RA^+^CCR7^-^CD94^+^CD57^+^ |
| C22 | CD8^+^ effector T cells | CD3^+^CD8a^+^CD45RA^+^CCR7^-^CD56^+^ |
| C23 | CD8^+^ effector T cells | CD3^+^CD8a^+^CD45RA^+^CCR7^-^CD57^+^ |
| C24 | γδT cells | CD3^+^CD4^-^CD8a^-^CD56^+^CD11c^+^CD57^+^ |
| C25 | γδT cells | CD3^+^CD4^-^CD8a^-^CD161^+^CD57^+^ |
| C26 | Undefined | CD45^low^CD16^+^ |
| C27 | NK cells | CD56^+^CD16^+^CD11b^+^CD57^-^CD38^+^ |
| C28 | NK cells | CD56^+^CD16^+^CD11b^-^CD8a^+^CD38^-^CD161^-^ |
| C29 | Basophils | CD123^+^HLA-DR^-^CD11b^+^CD33^+^ |
| C30 | Basophils | CD123^+^HLA-DR^-^CD11b^+^CD33^-^ |
| C31 | CD11c^+^ B cells | CD19^+^HLA-DR^+^CD11c^+^CD20^+^ |
| C32 | Naive B cells | CD19^+^HLA-DR^+^IgD^+^CD27^-^CD24^+^CXCR5^+^CD38^+^CD20^+^ |
| C33 | Memory B cells | CD19^+^HLA-DR^+^IgD^-^CD27^+^CD24^+^CXCR5^+^CD38^-^CD20^+^ |
| C34 | Plasmablasts | CD19^+^HLA-DR^+^IgD^-^CD27^+^CD38^+^CD24^-^CD86^+^CD20^-^ |
| C35 | Non-classical monocytes | CD14^low^CD16^+^HLA-DR^+^CD11b^+^ |
| C36 | Classical monocytes | CD14^+^CD16^low^HLA-DR^+^CD11b^+^ |
| C37 | mDC | CD11c^+^HLA-DR^+^CD33^+^CD38^+^CD39^+^ |
|  |  |  |

# 2 Supplementary Table 2

**Classification of PBMC-derived immune cell subsets based on surface markers**

| Lineage | cell subset | cluster |
| --- | --- | --- |
| γδ T cell | γδ T cell | C01, C04, C20, C24, C25 |
| Monocyte | Classical monocyte | C36 |
|  | Non-classical monocyte | C35 |
| NK cell | NK cell | C18, C19, C27, C28 |
| CD8^+^T cell | CD8^+^Teff | C02, C03, C21, C22, C23 |
|  | CD8^+^Tem | C05, C06 |
| CD4^+^T cell | Naïve CD4^+^ T | C07 |
|  | CD4^+^Tem | C08, C11, C12, C13, C14, C16, C17 |
|  | CD4^+^Teff | C15 |
|  | CD4^+^Tcm | C09, C10 |
| B cells | Naïve B cells | C32 |
|  | Memeory B cells | C33 |
|  | Plasmablasts | C34 |
|  | CD11c^+^ B cells | C31 |
| Basophils | Basophils | C29, C30 |
| mDC | mDC | C37 |
| Undefined | Undefined | C26 |
|  |  |  |

# ( Tem, effector memory T cells; Tcm, central memory T cells; Teff, effector T cells )

# 3 Supplementary Table 3

**Antibody Cocktail Table**

| **No.** | **Mass tag** | **Target** | **Clone** | **Working Concentration (ng/μL)** | **Vendor** |
| --- | --- | --- | --- | --- | --- |
| 1 | 89Y | CD45 | HI30 | 2 | BioLegend |
| 2 | 115ln | CD3 | UCHT1 | 1 | Bio cell |
| 3 | 141Pr | CD56 | NCAM16.2 | 0.25 | BD biosciences |
| 4 | 142Nd | TCRgd | 5A6.E9 | 1 | Thermofisher |
| 5 | 143Nd | CD196(CCR6) | G034E3 | 2 | BioLegend |
| 6 | 144Nd | CD14 | M5E2 | 4 | BioLegend |
| 7 | 145Nd | IgD | IA6-2 | 0.5 | BioLegend |
| 8 | 146Nd | CD123(IL-3R) | 6H6 | 0.5 | BioLegend |
| 9 | 147Sm | CD85j | GHI/75 | 5 | BioLegend |
| 10 | 148Nd | CD19 | HIB19 | 1 | BioLegend |
| 11 | 149Sm | CD25(IL-2R) | 24212 | 0.5 | R&D |
| 12 | 150Nd | CD274(PD-L1) | 29E.2A3 | 0.5 | BioLegend |
| 13 | 151Eu | CD278(ICOS) | C398.4A | 1 | BioLegend |
| 14 | 152Sm | CD39 | A1 | 2 | BioLegend |
| 15 | 153Eu | CD27 | O323 | 1 | BioLegend |
| 16 | 154Sm | CD24 | ML5 | 4 | BioLegend |
| 17 | 155Gd | CD45RA | HI100 | 0.5 | BioLegend |
| 18 | 156Gd | CD86 | Fun-1 | 4 | BD biosciences |
| 19 | 157Gd | CD28 | CD28.2 | 8 | BioLegend |
| 20 | 158Gd | CD197(CCR7) | G043H7 | 4 | BioLegend |
| 21 | 159Tb | CD11c | BU15 | 0.5 | BioLegend |
| 22 | 160Gd | CD33 | WM53 | 0.25 | BioLegend |
| 23 | 161Dy | CD152(CTLA-4) | 14D3 | 4 | eBioscience |
| 24 | 163Dy | CD161 | HP-3G10 | 4 | BioLegend |
| 25 | 164Dy | CD185(CXCR5) | RF8B2 | 0.5 | BD biosciences |
| 26 | 165Ho | CD66b | G10F5 | 0.5 | BioLegend |
| 27 | 166Er | CD183(CXCR3) | G025H7 | 2.5 | BioLegend |
| 28 | 167Er | CD94 | HP-3D9 | 0.25 | BD biosciences |
| 29 | 168Er | CD57 | HNK-1 | 0.25 | BioLegend |
| 30 | 169Tm | CD45RO | UCHL1 | 2 | BioLegend |
| 31 | 170Er | CD127(IL-7Ra) | A019D5 | 2 | BioLegend |
| 32 | 171Yb | CD279(PD-1) | EH12.2H7 | 2 | BioLegend |
| 33 | 172Yb | CD38 | HIT2 | 2 | BioLegend |
| 34 | 173Yb | CD194(CCR4) | L291H4 | 0.5 | BioLegend |
| 35 | 174Yb | CD20 | 2H7 | 1 | BioLegend |
| 36 | 175Lu | CD16 | 3G8 | 2 | BioLegend |
| 37 | 176Yb | HLA-DR | L243 | 2 | BioLegend |
| 38 | 197Au | CD4 | RPA-T4 | 0.5 | BioLegend |
| 39 | 198Pt | CD8a | RPA-T8 | 0.25 | BioLegend |
| 40 | 209Bi | CD11b | M1/70 | 0.25 | BioLegend |

**4 Supplementary Detailed Methods (CyTOF)**

1. Sample preparation(Blood sample)

EDTA-treated whole blood was separated PBMC by Ficoll density gradient centrifugation. 5 mL cold FACS Buffer (1×PBS+0.5%BSA) was added to resuspend the pellet. The cells were collected by centrifugation at 400g for 5 minutes at 4℃, resuspended in FACS Buffer after supernatant aspirated and then counted. At least 3 million cells per sample and over 85% viability were required for the quality control of blood sample.

2. Cell staining

3 million cells were aliquoted into 1.5 ml polystyrene tubes for each sample to be stained. In the preparation of Cell-ID Cisplatin Solution (final concentration of 250 nM 194Pt) in PBS, cells were resuspended in 100 μL Cisplatin Solution and incubated for 5 minutes on ice, then washed twice after adding 1 mL FACS Buffer to each tube, centrifuged at 400 g for 5 min at 4℃ and discarded supernatant by aspiration. Tubes were next incubated for 20 minutes on ice with Fc-receptor blocking solution added to each of them. Quality control requirements of metal-tagged antibodies: The antibody was titrated with relevant conditions and positive cells to validate and optimize noise-signal ratio. After the preparation of Antibody Cocktail in FACS Buffer (See Supplementary Table 3: Antibody Cocktail Table), each tube, added with 50 μL the antibody cocktail, was incubated for 30 minutes on ice. Following the incubation, cells were washed twice by adding 1 mL FACS Buffer to each tube, centrifuged at 400g for 5 minutes at 4 ℃ and discarded supernatant by aspiration. After the preparation of cell intercalation in Maxpar Fix and Perm Buffer (final concentration of 250 nM 191/193Ir), 200 μL of the cell intercalation solution was added into each tube, gently vortexed and left overnight at 4℃. After washed with PBS, we use unique barcoding isotope combination to label individual cell samples for 30 minutes. Cells were washed sequentially by 1 ml FACS Buffer and 2 ml of deionized water, centrifuged at 800g for 5 minutes at 4℃ and discarded supernatant by aspiration respectively. Next, these cells were resuspended in 1-2 mL deionized water and then counted. In this case, samples are ready for CyTOF.

3. Data Acquisition

The CyTOF was turned on to enable ensemble sample be introducted into system. To calibrate the CyTOF, a Tuning and QC procedures were ran, using Tuning solution and EQ beads. Cells were resuspended and adjusted to 1×10^6^/ml with deionized water containing 20% EQ beads. Afterwards, these cells were transferred through a 40 μm filter to a new FACS tube so data could be acquired on the CyTOF system.

4. Data Analysis

Data of each sample were debarcoded from raw data using a doublet-filtering scheme with unique mass-tagged barcodes. Each .fcs file generated from different batches were normalized through bead normalization method. FlowJo software was used to manipulated gate data manually to exclude debris, dead cells and doublets, leaving live, single immune cells. The PhenoGraph clustering algorithm was applied to all cells in order to partition the cells into distinct phenotypes based on marker expression levels. Clustering parameters were adjusted to obtain suitable number of clusters. Cell types of each cluster were annotated according to its marker expression pattern on a heatmap of cluster vs marker. The dimensionality reduction algorithm t-SNE was used to visualize the high-dimensional data in two dimensions, showing the distribution of each cluster and the expression of the markers, as well as the differences between groups or different sample types. T-test statistical analysis was performed on the frequency of annotated cell population. Also, manually gate was performed to obtain major immune cell population, like T and B lymphocytes, NK cells, and expression level of functional markers.

**5 Supplementary Detailed Methods (Flow cytometry)**

1. Isolation of PBMC from peripheral blood

3-5ml of peripheral blood samples were isolated for PBMC within 6 hours of collection. Three 15mL sterile centrifuge tubes were taken for each sample, labelled as tubes 1, 2 and 3 (note: tube 1 was used for blood sample dilution, tube 2 for lymphocyte isolation and tube 3 for lymphocyte washing after isolation). Pour each blood sample into a separate No. 1 centrifuge tube, dilute and mix according to equal volume. Then 3mL Ficoll cell separation solution was added to the bottom of the No. 2 centrifuge tube for each sample, and the diluted blood sample was carefully spread on top of the separation solution and centrifuged at 400g/min for 30min at room temperature. After centrifugation the liquid is clearly divided into 4 layers from top to bottom. The first layer is the plasma, the second layer is the PBMC, the third layer is the cell isolate and the fourth layer is the red cells. Immediately afterwards, the second layer of PBMC was aspirated into a No. 3 centrifuge tube using a barrel, 5-7 mL of serum-free medium was added and the cells were resuspended, centrifuged at 350-500 g/min for 5 min and the supernatant was discarded, and this procedure was repeated once. The cell precipitate was then resuspended in RPMI-1640 medium complete with 10% fetal bovine serum and counted using a cell counting plate.

2. Cell culture and stimulation

Configure cell culture medium consisting of 9 mL of 1640 medium, 1 mL of FBS, and 20 μL of Cell Activation Cocktail (with Brefeldin A) per 10 ml of cell culture medium. Dilute the cell density to 1 × 10^6^cells/mL with the prepared cell culture medium. Take 1mL of cell suspension (1×10^6^) into a 24-well plate, and incubate the plate in a 37°C, 5% CO2 cell incubator for 4-6h (maximum 12h). Remove the cell culture plates from the cell incubator and collect the cells from the in vitro stimulated culture, centrifuge at 350 g/min for 5 min and discard the supernatant.

3. Cell Labeling and Flow cytometry

Cells were resuspended using 100 μL of PBS and stained in the order of dead and live cell labelling, surface antibody labelling,intracellular and nuclear labelling. Fixation and membrane breaking was performed using the Fixation Breaking Kit prior to intracellular labelling. After staining is complete, cells are resuspended with 200-300 ul PBS and flow cytometry was performed.

All reagents were purchased from BD biosciences：Fixable Viability Stain 780 (Cat. 565388), CD3 PerCP(Cat. 552852), CD4 BB515 (Cat.564419), IFN-γBV510（Cat.563287），IL-17A BV421 (Cat.562933), CD25 PE (Cat.555432)，FoxP3 Alexa 647 (Cat.560045), Transcription Factor Buffer Set (Cat.562574), Leukocyte Activation Cocktail, with BD GolgiPlug™ (Cat.550583), Fixation Breaking Kit (BD: 554714).

**6 Supplementary Detailed Methods (Microarray datasets)**

In this study, the raw gene expression profiles GSE59071 was downloaded from the GEO database (https://www.ncbi.nlm.nih.gov/geo/). The GSE59071 expression profile consists of 11 healthy control colon, 23 remission colon and 82 active colon, detected by the Affymetrix Human Genome U133 Plus 2.0 Array.
